# Common Infectious Agents and Monoclonal B-Cell Lymphocytosis: A Cross-Sectional Epidemiological Study among Healthy Adults

**DOI:** 10.1371/journal.pone.0052808

**Published:** 2012-12-28

**Authors:** Delphine Casabonne, Julia Almeida, Wendy G. Nieto, Alfonso Romero, Paulino Fernández-Navarro, Arancha Rodriguez-Caballero, Santiago Muñoz-Criado, Marcos González Díaz, Yolanda Benavente, Silvia de Sanjosé, Alberto Orfao, the Primary Health Care Group of Salamanca for the Study of MBL

**Affiliations:** 1 Unit of Infections and Cancer (UNIC), IDIBELL, Institut Català d’ Oncologia, L’ Hospitalet de Llobregat, Barcelona, Spain; 2 CIBER Epidemiología y Salud Pública (CIBERESP), Barcelona, Spain; 3 Instituto de Biologia Molecular y Celular del Cancer, Centro de Investigacion del Cancer/IBMCC (CSIC-USAL), IBSAL, Department of Medicine and Cytometry Service, University of Salamanca, Salamanca, Spain; 4 Gerencia de Atención Primaria de Salud, Centro de Atención Primaria de Salud Miguel Armijo Salamanca, Sanidad de Castilla y León (SACYL), Castilla y León, Spain; 5 Centro de Atención Primaria de Salud de Ledesma, Salamanca, Sanidad de Castilla y León (SACYL), Castilla y León, Spain; 6 Service of Microbiology, University Hospital of Salamanca, Salamanca, Spain; 7 Service of Hematology, University Hospital of Salamanca, IBMCC, IBSAL and Department of Medicine, University of Salamanca, Salamanca, Spain; DRFZ, Germany

## Abstract

**Background:**

Risk factors associated with monoclonal B-cell lymphocytosis (MBL), a potential precursor of chronic lymphocytic leukaemia (CLL), remain unknown.

**Methods:**

Using a cross-sectional study design, we investigated demographic, medical and behavioural risk factors associated with MBL. “Low-count” MBL (cases) were defined as individuals with very low median absolute count of clonal B-cells, identified from screening of healthy individuals and the remainder classified as controls. 452 individuals completed a questionnaire with their general practitioner, both blind to the MBL status of the subject. Odds ratios (OR) and 95% confidence interval (CI) for MBL were estimated by means of unconditional logistic regression adjusted for confounding factors.

**Results:**

MBL were detected in 72/452 subjects (16%). Increasing age was strongly associated with MBL (P-trend<0.001). MBL was significantly less common among individuals vaccinated against pneumococcal or influenza (OR 0.49, 95% confidence interval (CI): 0.25 to 0.95; P-value = 0.03 and OR: 0.52, 95% CI: 0.29 to 0.93, P-value = 0.03, respectively). Albeit based on small numbers, cases were more likely to report infectious diseases among their children, respiratory disease among their siblings and personal history of pneumonia and meningitis. No other distinguishing epidemiological features were identified except for family history of cancer and an inverse relationship with diabetes treatment. All associations described above were retained after restricting the analysis to CLL-like MBL.

**Conclusion:**

Overall, these findings suggest that exposure to infectious agents leading to serious clinical manifestations in the patient or its surroundings may trigger immune events leading to MBL. This exploratory study provides initial insights and directions for future research related to MBL, a potential precursor of chronic lymphocytic leukaemia. Further work is warranted to confirm these findings.

## Introduction

Monoclonal B-cell lymphocytosis (MBL) is defined by the presence of <5×10^9^ clonal B-cells/L in peripheral blood (PB) of healthy individuals [1], [2]. Two entities can be distinguished within MBL, based on the absolute count of clonal B-cells: a) those diagnosed in clinical settings and associated with lymphocytosis with a median absolute count of clonal B-cells >1.5×10^9^/L; and b) population-screened MBL, the so-called “low-count” MBL, with very low median absolute count of clonal B-cells of about 0.05×10^9^/L, identified in population-screening studies of healthy individuals using high-sensitive flow cytometry approaches [3], [4]. Based on immunophenotypic grounds, MBL can be classified as chronic lymphocytic leukaemia (CLL)-like MBL, with a CD5^+^, CD23^+^ and CD20^low^ phenotype representing the most common subgroup (∼ 75/80% of MBL), atypical-CLL (CD5^+^, CD20^bright^) and CD5^−^ MBL [2]. With advanced flow cytometry techniques, low count “CLL-like” MBL is detected in 12%–14% [5], [6] of healthy adults in population-screening studies. Recent research suggests systematic occurrence of clinical CLL-like MBL prior to CLL [7]. However, most CLL-like MBL patients never develop clinical complications and the estimated yearly rate of progression of clinical CLL-like MBL to CLL with treatment requirement is 1–2% [8]; in turn, the rate of progression of “low count” CLL-like MBL is still unknown.

The aetiology of MBL and CLL remains unknown and few studies have been reported on potential risk factors for MBL. Unambiguous risk factors associated to both CLL and MBL are increasing age [9] and genetic susceptibility [10]. In turn, male predominance is also recurrently reported in CLL but results on MBL are controversial [11]. Exposure to pesticide, herbicides and chemical agents has also been associated with CLL [12]. Caucasian ethnicity has long stood as a risk factor for CLL with lower incidence rates among Asian than Caucasian Americans. However, recent studies reported higher CLL incidence rates among Asian US born than Asian foreign born [13] subjects and increasing trends in CLL incidence rates in Taiwan [14]. Altogether, these results suggest a potential role for some strong but unidentified environmental factors in the aetiology of CLL. Recently, Moreira et al. (2012) reported that the risk of hospitalisation for infections was more common in newly diagnosed clinical MBL and CLL patients than controls [15]. Other risk factors associated with MBL might include living near a hazardous waste site [11].

As part of a study examining the prevalence of MBL in the general population [5], [6], we investigated potential risk factors associated with “low count” MBL using a cross-sectional study design among 452 healthy subjects randomly selected from the Primary Health Care system of the region of Salamanca (Spain). This exploratory study provides initial insights and directions for future research related to MBL, a potential precursor of chronic lymphocytic leukaemia. In particular our findings suggest that exposure to infectious agents leading to serious clinical manifestations in the patient or its surroundings may trigger immune events leading to MBL.

## Methods

### Design and Subjects

As part of a study examining the prevalence of MBL through highly sensitive multicolor flow cytometry in a cohort of 639 healthy adults with normal PB lymphocyte counts from the general population of the Primary Health Care system region of Salamanca (northwest-central Spain) [5], we investigated risk factors associated with “low-count” MBL. All these 639 cases have been described previously in clinical, as well as phenotypic/genetic and molecular terms [5], [6]. Among the 639 subjects older than 40 years, 452 (71%) completed a questionnaire with their general practitioner, both blind to the MBL status of the subject. Individuals with MBL were denoted as cases and the remainder classified as controls. MBL cases were further classified as either “CLL-like” or non-“CLL-like” MBL, based on the presence *versus* absence of a CD5+, CD23+ and CD20^dim^ immunophenotype, respectively. The research protocol was approved by the Ethics Committee of the Cancer Research Center of Salamanca and all participants gave their written informed consent in accordance with the Declaration of Helsinki.

### Immunophenotypic Analyses

The flow cytometry approach has been described in detail in the previous study [5]. In brief, per case, between 1 and 4 mL of EDTA-anticoagulated PB was immunophenotyped using a highly sensitive 8-color flow cytometry. The minimum number of clustered cellular events required to define the presence of a clonal B-cell population was 50.

### Statistical Analyses

All statistical analyses were performed using STATA10.1 (Statacorp, USA). We used unconditional logistic regression adjusted for age (<50, 50–59, 60–69, 70 or more) and sex to calculate odds ratios (OR) and 95% confidence intervals (CI) for MBL in relation to different risk factors from the questionnaire. Further adjustment for family size, number of children or number of siblings was performed as appropriate (for instance, the variable self-reported history of infections among children was further adjusted for number of children). Since pneumococcal and influenza vaccinations are generally provided from the age of 60 years in Spain, further analyses stratified by age (≥60 years) were performed. Self-reported current drug use was grouped into 14 major groups of drugs as per the Anatomical Therapeutic Chemical Classification System (2003). Sensitivity analysis restricting the outcome to CLL-like MBL was performed.

## Results

Overall, 72/452 subjects (16%) were diagnosed with “low-count” MBL (mean absolute B-cell count: 0.055; standard deviation: 0.216; maximum: 1.172×10^9^ B-cells/L) (Table 1). Most cases (60/72; 83%) were classified as CLL-like MBL. About half of the cases (48%) and the controls (49%) were males (P = 0.9). OR of “low-count” MBL cases increased with increasing age (P<0.001). Cases and controls did not differ in terms of area of recruitment, residence of birth, tobacco and alcohol consumption, body mass index, height, weight and women reproductive history (data not shown).

**Table 1:** Socio-demographic and descriptive characteristics of “low-count” monoclonal B-cell lymphocytosis (MBL) cases and non-MBL subjects (controls).

	Controls N = 380	MBL cases N = 72	OR^1^ & 95% CI
**SEX**			
Male	181 (48%)	35 (49%)	Ref
Female	199 (53%)	37 (51%)	1.04 (0.62 to 1.75)
			P-het = 0.9
**AGE**			
<50	92 (24%)	6 (8%)	Ref
50–59	99 (26%)	7 (10%)	1.09 (0.35 to 3.35)
60–69	78 (21%)	19 (26%)	3.74 (1.42 to 9.84)
70–79	85 (22%)	26 (36%)	4.70 (1.84 to 12.00)
80 or more	26 (7%)	14 (19%)	8.26 (2.89 to 23.62)
			P-trend<0.0001
mean (SD)	60 (13)	70 (11)	
range	40 to 97	43 to 95	
**AREA OF RECRUITMENT**			
Salamanca suburb	64 (17%)	8 (11%)	Ref
Salamanca centre	95 (25%)	19 (26%)	1.50 (0.60 to 3.73)
Rural	221 (58%)	45 (63%)	1.46 (0.64 to 3.34)
			*P-het = 0.6*
**RESIDENCE AT BIRTH**			
Salamanca City	221 (59%)	40 (57%)	Ref
Salamanca county, excluding Salamanca city	97 (26%)	23 (33%)	1.11 (0.62 to 1.99)
Other Spanish counties	45 (12%)	7 (10%)	0.95 (0.39 to 2.31)
Other country	9 (2%)	0 (0%)	NA
			*P-het = 0.9*
**TOBACCO CONSUMPTION**			
Never	220 (58%)	48 (67%)	Ref
Past	90 (24%)	15 (21%)	0.86 (0.40 to 1.85)
Current	69 (18%)	9 (13%)	1.08 (0.44 to 2.65)
			*P-het = 0.9*
**ALCOHOL CONSUMPTION**			
Never	212 (56%)	42 (60%)	Ref
2–4 times/week	50 (13%)	7 (10%)	0.92 (0.37 to 2.32)
Week-end	56 (15%)	4 (6%)	0.57 (0.18 to 1.77)
Everyday	58 (15%)	17 (24%)	1.57 (0.72 to 3.43)
			*P-het = 0.2*
**BODY MASS INDEX, kg/cm^2^**			
<25	84 (29%)	15 (27%)	Ref
25–29	129 (45%)	23 (42%)	0.88 (0.42 to 1.85)
≥30	75 (26%)	17 (31%)	1.00 (0.45 to 2.22)
			*P-trend = 1.0*
mean (SD)	27.6 (4.6)	27.8 (4.0)	
* missing*	*92 (24%)*	*17 (24%)*	

1 Adjusted for age (<50, 50–59, 60–69, 70+) and sex.

Ref: reference group; N: number; SD: standard deviation; het: heterogeneity.

OR: Odds ratio; CI: confidence interval.

**Figure 1:**
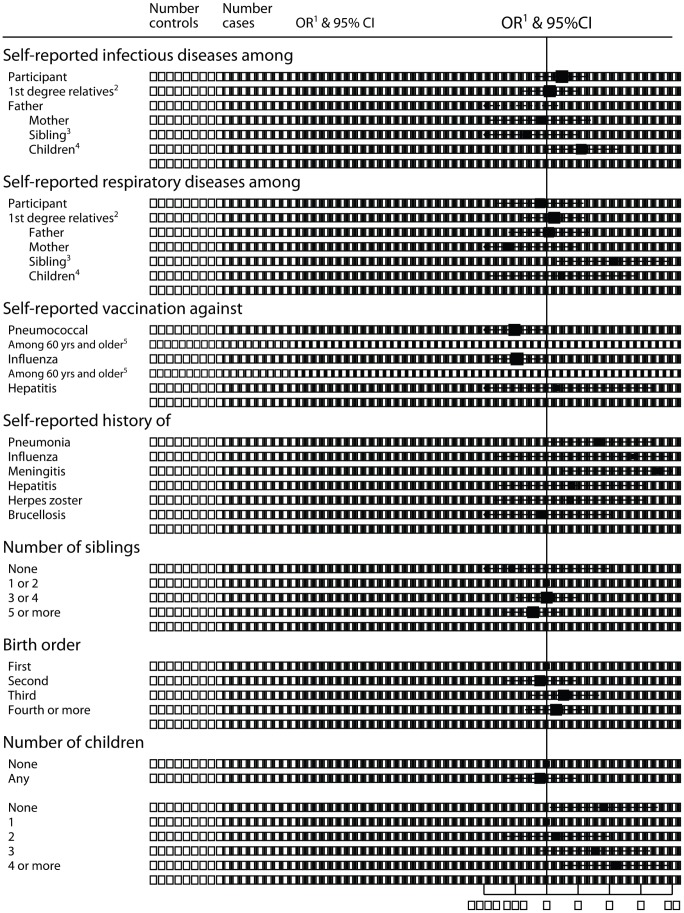
Odds ratios for “low-count” monoclonal B-cell lymphocytosis by selected potential variables related to infectious agents. Ref: reference group; N: number; het: heterogeneity; OR: Odds ratio; CI: confidence interval ^1^: Adjusted for age (<50, 50–59, 60–69, 70+) and sex ^2^: Further adjusted for family size (using the total number of children and siblings; categories: <6; 6 or more; missing). ^3^: Further adjusted for number of siblings (categories: <2; 3 or more; missing) *^4^:* Further adjusted for number of children (0/2; 3or more; missing) *^5^:* Adjusted for age (<70, 70+) and sex, N_controls_ = 189; N_cases_ = 59 P-values were calculated using Wald-test. Black squares indicate OR, the area of each square being proportional to the amount of statistical information contributed. Horizontal lines represent 95% CI.

Conversely, a clear association with transmission and exposure to infection agents was found (Figure 1). In detail, “low-count” MBL cases were less likely to have reported having pneumococcal (OR: 0.49; 95% CI: 0.25 to 0.95; P = 0.03) and influenza (OR: 0.52; 95% CI: 0.29 to 0.93; P = 0.03) vaccination and more likely to have had pneumonia (OR: 3.26; 95% CI: 1.03 to 10.27; P = 0.04), meningitis (OR: 11.73; 95%CI: 1.45 to 95.13; P = 0.02) or influenza (OR: 6.72; 95% CI: 0.31 to 146.70; P = 0.2). Albeit based on small numbers, such association was also supported by an increased number of reported infectious diseases in the children of cases (OR: 2.14; 95%: 0.92 to 5.01; P = 0.08) and of respiratory diseases among their siblings (OR: 4.35; 95% CI: 1.23 to 15.34; P = 0.02). Furthermore, the OR for “low-count” MBL increased with increasing number of children among cases with children (P<0.001), such trend being observed separately in men and women (P<0.001). However, childless individuals were also three times more likely to have been diagnosed with MBL compared to individuals with only 1 child and no potential confounders could explain this association.

MBL cases were also more likely to report haematological cancer and solid cancer among their first-degree relatives, compared to controls (Table 2). Conversely, cases and controls did not differ in terms of the current use of any of the 14 different groups of drugs. However, 6% (N = 4) of “low-count” MBL cases versus 12% (N = 44) of controls (OR: 0.30; 95% CI: 0.10 to 0.87; P = 0.03), reported treatment for diabetes (Table S1 in File S1). Noteworthy, all associations described above were retained when the analyses were restricted to CLL-like MBL (Table 3). MBL load was not associated with any of the exposure variables, whereas normal B-cell count was decreasing with increasing age and was higher in women than men (data not shown).

**Table 2:** Odds ratios (OR) estimates, with 95% confidence intervals (CI), for “low-count” monoclonal B-cell lymphocytosis by self-reported family history of cancer.

	Controls N = 380	MBL cases N = 72	OR^1^ & 95% CI
**Ever had family history of haematological cancer**			
None	356 (94%)	63 (87%)	ref
1 family member affected^2^	22 (12%)	8 (28%)	1.96 (0.79 to 4.86)
2 family members affected^2^	2 (1%)	1 (3%)	3.70 (0.28 to 49.35)
			*P-trend = 0.07*
Ever (any family members)^2^	24 (6%)	9 (13%)	2.07 (0.87 to 4.93); *P = 0.1*
Participant	1 (<1%)	0 (0%)	NA
1^st^ degree relatives^2^	22 (6%)	9 (13%)	2.23 (0.93 to 5.38); *P = 0.07*
* Father*	*4 (1%)*	*4 (6%)*	11.49 (2.42 to 54.55); *P = 0.002*
* Mother*	*4 (1%)*	*0 (0%)*	*NA*
* Sibling* 3	*11 (3)*	*6 (8)*	2.46 (0.84 to 7.17); *P = 0.1*
* Children* 4	*4 (1%)*	*0 (0%)*	*NA*
2^nd^ degree relatives	2 (<1%)	0 (0%)	*NA*
**Ever had family history of solid cancer**			
None	168 (44%)	26 (36%)	
1 family member affected^2^	145 (38%)	32 (44%)	1.57 (0.87 to 2.84)
2 family members affected^2^	48 (13%)	9 (13%)	1.57 (0.87 to 2.84)
3 family members affected^2^	18 (5%)	4 (6%)	1.66 (0.49 to 5.65)
4 family members affected^2^	1 (<1%)	1 (2%)	9.87 (0.42 to 232.60)
			*P-trend = 0.2*
Ever (any family members)^2^	212 (56%)		1.54 (0.89 to 2.66); *P = 0.1*
Participant	27 (7%)		0.83 (0.32 to 2.15); *P = 0.7*
1^st^ degree relatives^2^	164 (43%)		2.02 (1.17 to 3.47); *P = 0.01*
* Father*	87 (23%)		1.03 (0.54 to 1.97); *P = 0.9*
* Mother*	57 (15%)		0.86 (0.38 to 1.96); *P = 0.7*
* Sibling* 3	60 (16%)		2.92 (1.59 to 5.36); P = 0.001*
* Prostate* ^,^ 5	*6 (2%)*		*6.31 (1.99 to 20.00); P = 0.002*
* Children* 4	6 (2%)		1.56 (0.28 to 8.59); *P = 0.6*
2^nd^ degree relatives	62 (16%)		0.95 (0.39 to 2.32); *P = 0.9*
**Ever had family history of solid and/or haematological cancers** 2			
Never	156 (41%)	20 (28%)	ref
With family history of solid cancer only	200 (53%)	43 (60%)	1.89 (1.04 to 3.45)
With family history of haematological cancer only	12 (3%)	6 (8%)	4.23 (1.33 to 13.50)
With family history of both type of cancers	12 (3%)	3 (4%)	1.93 (0.46 to 8.09)
			*P-heterogeneity = 0.02*

Ref: reference group; N: number; NA: not estimated; het: heterogeneity.

OR: Odds ratio; CI: confidence interval; 1^st^ degree relatives: parents, siblings and children; 2^nd^ degree relatives: grand-parents.

1 Adjusted for age (<50, 50–59, 60–69, 70+) and sex.

2 Further adjusted for family size (using the total number of children and siblings; categories: <6; 6 or more; missing).

3 Further adjusted for number of siblings (categories: <2; 3 or more; missing).

4 Further adjusted for number of children (<2; 3 or more; missing).

5 Adjusted for number of brothers (<3; 3 or more; missing).

**Table 3:** Odds ratios (OR) estimates, with 95% confidence intervals (CI), for “low-count” CLL-like monoclonal B-cell lymphocytosis (60 out of 72 cases) by previous associated factors.

	ControlsN = 380	MBL casesN = 60	OR^1^ & 95% CI
**Age**			
<50	92 (24%)	6 (10%)	Ref
50–59	99 (26%)	5 (8%)	0.78 (0.23 to 2.66)
60–69	78 (21%)	16 (27%)	3.19 (1.19 to 8.57)
70–79	85 (22%)	22 (37%)	4.06 (1.56 to 10.52)
80 or more	26 (7%)	11 (18%)	6.56 (2.21 to 19.45)
mean (SD)	60 (13)	69 (12)	
			*P-trend<0.0001*
range	40 to 97	43 to 93	
**Self-reported infectious diseases among**			
Children^2^	32 (8%)	8 (13%)	2.33 (0.95 to 5.69); P = 0.06
**Self-reported respiratory diseases among**			
Sibling^3^	7 (2%)	4 (7%)	4.32 (1.13 to 16.56); P = 0.03
**Self-reported vaccination against**			
Pneumococcus^4^	78 (41%)	14 (29%)	0.48 (0.24 to 0.99); P = 0.05
Influenza	153 (40%)	26 (43%)	0.58 (0.31 to 1.07); P = 0.08
**Self-reported respiratory tract infections**			
Pneumonia	8 (2%)	6 (10%)	4.18 (1.31 to 13.27); P = 0.02
Influenza	1 (<1%)	1 (2%)	7.49 (0.35 to 162.48); P = 0.2
Meningitis	3 (<1%)	1 (2%)	9.79 (0.80 to 119.99); P = 0.07
**Number of children**			
None	57 (15%)	14 (23%)	3.24 (0.96 to 10.85)
1	67 (18%)	4 (7%)	Ref
2	141 (37%)	11 (18%)	1.07 (0.32 to 3.61)
3	66 (18%)	11 (18%)	2.07 (0.59 to 7.32)
4 or more	46 (12%)	20 (33%)	4.23 (1.24 to 14.43)
			**P-trend (in parous)<0.0001**
**Diabetes treatment**	44 (12%)	3 (5%)	0.27 (0.08 to 0.91); *P = 0.04*
**Ever had family history of haematological cancer**			
Ever (any family members)^5^	24 (6%)	8 (13%)	2.18 (0.88 to 5.40); *P = 0.09*
1^st^ degree relatives^5^	22 (6%)	8 (13%)	2.36 (0.94 to 5.94); *P = 0.07*
* Father*	4 (1%)	4 (7%)	16.30 (3.30 to 80.58); *P = 0.001*
**Ever had family history of solid cancer**			
1^st^ degree relatives^5^	164 (43%)	34 (57%)	1.72 (0.97 to 3.08); *P = 0.07*
* Sibling* 4	60 (16%)	21 (35%)	*2.43 (1.25 to 4.73); P = 0.009*
* Prostate* 6	6 (2%)	6 (10%)	*5.95 (1.71 to 20.73); P = 0.005*

^1^Adjusted for age (<50, 50–59, 60–69, 70+) and sex.

2 Further adjusted for number of children (<2; 3 or more; missing).

3 Further adjusted for number of siblings (categories: <2; 3 or more; missing).

4 *Among 60 years old and older* ^,^ *N_controls_ = 189; N_cases_ = 49.*

5 Further adjusted for family size (using the total number of children and siblings; categories: <6; 6 or more; missing).

6 Further adjusted for number of brothers (<3; 3 or more; missing).

Ref: reference group; N: number; OR: Odds ratio; CI: confidence interval; SD: standard deviation.

## Discussion

To our knowledge, this is the first epidemiological study investigating risk factors associated with MBL in the general population. In contrast to CLL, the male predominance was not observed for “low-count” MBL in our data whereas, as expected, OR of cases increased with increasing age. Our main findings were that lifetime exposure to several infectious agents might be associated with MBL aetiology.

Assuming that MBL, in particular CLL-like subtype, is a precursor of CLL [7], our findings are consistent with previous studies on CLL reporting that a personal history of pneumonia was associated with subsequent development of CLL [16]–[18]. Recent data also showed an increased risk of hospitalisation for infections among newly diagnosed clinical MBL than controls [15]. Since general population “low-count” CLL-like MBL is 100 times more common than CLL among the elderly [19] and persists over time without clinical progression [4], it was therefore postulated that “low-count” CLL-like MBL are less likely than clinical CLL-like MBL to progress to CLL: among 76 examined patients (median age: 66 years; range: 25–92 years) with low-count population-screening CLL-like MBL, none developed CLL after a 3-year median follow-up, even if they were found to carry 13q deletion with the same frequency to that observed in patients with newly diagnosed CLL and clinical MBL [4]. Hence, “low-count” CLL-like MBL might even be a normal stage of the immunosenescence process [4]. The observed link with infections for “low-count” CLL-like MBL might appear of limited value if the rate of progression of “low-count” CLL-like MBL to CLL is very low; however this progression rate is still unknown and large prospective studies are needed to evaluate which patients with MBL will advance to CLL. The progression from CLL-like MBL of “low-count” or clinical populations to CLL might then be due to the occurrence in MBL cells of specific biological and molecular profiles [20], potentially combined with exposure to some unknown environmental risk factors. Several hypotheses have been advanced to explain an infectious route in CLL. Accordingly, recent reports [21] suggest that chronic and persistent antigenic stimulation may have a role in MBL and CLL aetiology and, respiratory tract infections could particularly be triggers for CLL development [16]. Hepatitis C virus has also been suggested as a potential candidate for dysregulation of the immune system with recent data showing that the three MBL subtypes are more frequent in HCV infected individuals than in the general population [22]. In our data, we could not distinguish between the different hepatitis and no statistical significant association was observed with overall self-reported history of hepatitis or vaccination against hepatitis.

We observed that family history of cancer in particular haematological cancers was more common in patients with MBL than controls. In the published literature, MBL is indeed more frequent in families with CLL cases, in particular among first-degree relatives of patients with familial CLL [23]. Unfortunately, we could not differentiate here the association by haematological subtypes as this information was unavailable. In relation to the use of diabetes treatment and chronic lymphocytic leukaemia, data in the literature is inconsistent; while some authors report an increased risk of CLL among diabetic patients [24], others suggest a lower risk of lymphoma [25] and other cancers [26] particularly when using metformin. Further studies are needed to clarify this finding.There are several limitations of this study. The questionnaire provided limited information on occupation (either job title or company type/name was reported). Hence, occupational exposure to contaminants, organic solvents, herbicides or infections could not be examined. Furthermore the origin and type (viral/bacterial and chronic/acute) of infections that occurred among the children and other family members were not specified. However, this type of data is clearly difficult to collect in epidemiological settings. Other potential limitations of our study are the lack of validation of self-reports of family history even though questionnaires were filled in with general practitioners, as well as the limited information on timing of infection relative to date of recruitment. Despite the fact that the number of cases in each stratum was relatively small for some categories and that the number of comparisons performed was high, our results point out a potential role of infectious agents in the development of “low-count” MBL in the general population, particularly of those involved in respiratory infections. However, a reverse causality effect that would result on detecting a more suppressed immune system among subjects with MBL or already in the pathway of CLL cannot be excluded, as decreased numbers of normal PB B-cells as well as CD4+CD8+ double-positive T-cells have been specifically reported in "low-count” MBL [27]. Recent findings showing that regulatory T-cells increase gradually from controls to “clinical” MBL to CLL [28], and that most “low-count” MBL subjects show T-cell clones especially among CD4+CD8+ T-cells [4] further support the hypothesis of an altered immune system of MBL patients. Further studies analyzing dysregulations of the immune system in MBL compared to controls are required. Although selection biases cannot be ruled out, the robustness of the study relies in that neither the interviewers nor the study subjects were aware of the MBL status. This small exploratory study provides initial insights and directions for future research. Further studies are needed to evaluate the association between MBL and CLL and to examine the role of infectious agents in the development and progression of both entities.

## Supporting Information

File S1 Table S1 and list of members of the Primary Health Care Group of Salamanca for the Study of MBL (List S1)
(DOC)
