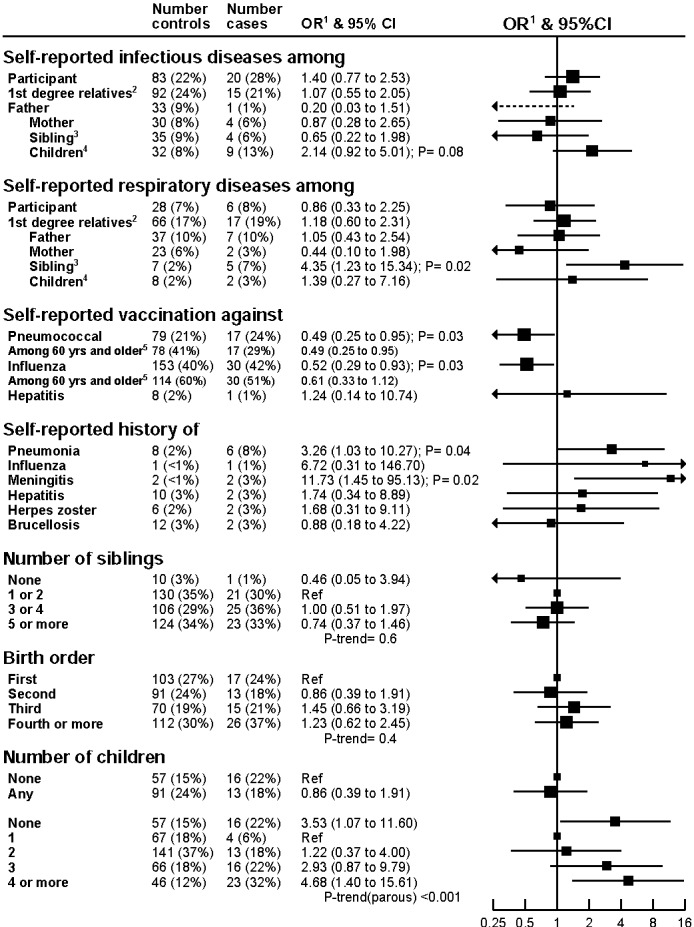# Correction: Common Infectious Agents and Monoclonal B-Cell Lymphocytosis: A Cross-Sectional Epidemiological Study among Healthy Adults

**DOI:** 10.1371/annotation/6f57f3c7-2f86-4d3a-a646-cb54de56ddba

**Published:** 2013-10-11

**Authors:** Delphine Casabonne, Julia Almeida, Wendy G. Nieto, Alfonso Romero, Paulino Fernández-Navarro, Arancha Rodriguez-Caballero, Santiago Muñoz-Criado, Marcos González Díaz, Yolanda Benavente, Silvia de Sanjosé, Alberto Orfao, the Primary Health Care Group of Salamanca for the Study of MBL

There was a rendering error which affected the readability of Figure 1. The correct Figure 1 can be viewed here: 

**Figure:**