# Supplementary material for: Common Infectious Agents and Monoclonal B-Cell Lymphocytosis: A Cross-Sectional Epidemiological Study among Healthy Adults
Source: PLoS One. 2012 Dec 28;7(12):e52808. doi: 10.1371/journal.pone.0052808 (PMC3532166; doi:10.1371/journal.pone.0052808)
Supplement: File S1 — Table S1 and list of members of the Primary Health Care Group of Salamanca for the Study of MBL (List S1) (DOC) [file pone.0052808.s001.doc]

**SUPPLEMENTARY MATERIAL**

**Table S1:** Odds ratios (OR) estimates, with 95% confidence intervals (CI), for “low-count” monoclonal B-cell lymphocytosis by self-reported use of drugs.

| **Drug family** | **Controls**  **N=380** | **MBL cases**  **N=72** | **OR¹ & 95% CI** |
| --- | --- | --- | --- |
| **Alimentary tract and metabolism**  *- Treatment of disorders caused by acids (A02)*  *- Diabetes (A10)*  *Insulin and analogues (A10A)*  *Hypoglycemic excluding insulin drugs (A10B)*  *Metformin (A10BA02)* | 123 (33%)  69 (18%)  44 (12%)  7 (2%)  42 (11%)  32 (9 %) | 25 (35%)  21 (29%)  4 (6%)  1 (1%)  4 (6%)  4 (6%) | 0.80 (0.46 to 1.40); *P=0.4*  1.49 (0.82 to 2.70); *P=0.2*  0.30 (0.10 to 0.87); *P=0.03*  0.49 (0.06 to 4.23); *P=0.5*  0.32 (0.11 to 0.94); *P=0.04*  0.45 (0.15 to 1.35); *P=0.2* |
| **Blood and blood forming organs** | 51 (14%) | 14 (20%) | 0.89 (0.44 to 1.77); *P=0.7* |
| **Cardiovascular system**  *- Statins (C10A)* | 168 (46%)  74 (19%) | 40 (56%)  22 (31%) | 0.84 (0.47 to 1.49); *P=0.5*  1.27 (0.71 to 2.30); *P=0.4* |
| **Dermatological** | 1 (<1%) | 0 (0) | NA |
| [**Genito-urinary system**](http://en.wikipedia.org/wiki/Genito-urinary_system) **and** [**sex hormones**](http://en.wikipedia.org/wiki/Sex_hormone) | 31 (8%) | 6 (8%) | 0.76 (0.29 to 1.98); *P=0.6* |
| **Systemic hormonal preparations, excluding sex hormones and insulins** | 25 (7%) | 3 (4%) | 0.70 (0.20 to 2.51); *P=0.6* |
| [**Anti-infectives**](http://en.wikipedia.org/wiki/Antiinfective) **for systemic use** | 4 (<1%) | 1 (1%) | 1.99 (0.20 to 19.91); *P=0.6* |
| **Antineoplastic and immunomodulating agents** | 9 (2%) | 3 (4%) | 2.16 (0.51 to 9.12); *P=0.3* |
| **Musculo-skeletal system**  *- Anti-inflammatory and anti-rheumatic products (M01)* | 89 (24%)  63 (17%) | 16 (23%)  8 (11%) | 0.85 (0.45 to 1.58); *P=0.6*  0.69 (0.31 to 1.54); *P=0.4* |
| **Nervous system**  *- Analgesic (N02)* | 118 (32%)  49 (13%) | 21 (30%)  14 (19%) | 0.79 (0.44 to 1.43); *P=0.4*  1.62 (0.79 to 3.30); *P=0.2* |
| **Antiparasitic products, insecticides and repellents** | 1 (<1%) | 0 (0%) | NA |
| **Respiratory system** | 26 (7%) | 5 (7%) | 0.83 (0.30 to 2.32); *P=0.7* |
| [**Sensory organs**](http://en.wikipedia.org/wiki/Sensory_organ) | 13 (4%) | 2 (3%) | 0.56 (0.12 to 2.61); *P=0.5* |
| **Various** | 0 (0) | 0 (0) | NA |

¹: Adjusted for age (<50, 50-59, 60-69, 70+) and sex.

N: number; NA: not estimated. OR: Odds ratio; CI: confidence interval

**List S1**

***Members of The Primary Health Care Group of Salamanca for the Study of MBL:***

| ALONSO MARTÍN MARIA MONSERRAT | C.S. FUENTES DE OÑORO |
| --- | --- |
| ASENSIO OLIVA MARIA CARMEN | C.S. SANTA MARTA DE TORMES |
| BAREZ HERNANDEZ PILAR | C.S. GARRIDO SUR |
| CARREÑO LUENGO MARIA TERESA | C.S. LEDESMA |
| CASADO ROMO JOSE MARIA | C.S. ALBA DE TORMES |
| CUBINO LUIS ROCIO | C.S. SANCTI SPIRITUS |
| DE VEGA PARRA JOSÉ | C.S. PEÑARANDA |
| FRANCO ESTEBAN ELOY | C.S. PIZARRALES-VIDAL |
| GARCÍA GARCÍA MARIA CONCEPCIÓN | C.S. GUIJUELO |
| GARCÍA RODRÍGUEZ BERNARDO LUCIO | C.S. LA ALBERCA |
| GARZON MARTIN AGUSTIN | C.S. PEÑARANDA |
| GOENAGA ANDRÉS ROSARIO | C.S. LEDESMA |
| GÓMEZ CABRERA ROSALIA | C.S. GARRIDO SUR |
| GOMEZ SANCHEZ FRANCISCO | C.S. PERIURBANA NORTE |
| GONZÁLEZ MORENO JOSEFA | C.S. GUIJUELO |
| GUARIDO MATEOS JOSE MANUEL | C.S. VITIGUDINO |
| HERNÁNDEZ SÁNCHEZ MARIA JESÚS | C.S. VITIGUDINO |
| HERRAES MARTÍN RICARDO | C.S. LA ALBERCA |
| JIMENEZ RUANO MARIA JOSEFA | C.S. GARRIDO NORTE |
| JIMENO CASCON TERESA BASA | C.S. ELENA GINEL DIEZ (TEJARES) |
| MACÍAS KUHN FRANCISCO | C.S. LEDESMA |
| MERINO PALAZUELO MIGUEL | C.S. FUENTES DE OÑORO |
| MIGUEL LOZANO RUBEN | C.S. GARRIDO NORTE |
| MONTERO LUENGO JUAN | C.S. SAN JUAN |
| MURIEL DIAZ Mª PAZ | C.S. MIGUEL ARMIJO |
| PABLOS REGUEIRO ARACELI | C.S. VITIGUDINO |
| PASTOR ALCALÁ LUIS | C.S. VITIGUDINO |
| PÉREZ DÍAZ MANUEL | C.S. PIZARRALES-VIDAL |
| PÉREZ GARCÍA MANUEL | C.S. ALBA DE TORMES |
| PRIETO GUTIERREZ Mª TERESA | C.S. PEÑARANDA |
| RAMOS MONGUE AURORA ESTHER | C.S. LEDESMA |
| RODRÍGUEZ MEDINA ANA MARIA | C.S. ALBA DE TORMES |
| RODRÍGUEZ VEGAS MARGARITA | C.S. LEDESMA |
| ROMO CORTINA JAVIER | C.S. ELENA GINEL DIEZ(TEJARES) |
| ROSELLÓ CARMEN ELENA | C.S. VITIGUDINO |
| SÁNCHEZ ALONSO BEGOÑA | C.S. ALDEADAVILA DE LA RIBERA |
| SÁNCHEZ BAZO BEGOÑA | C.S. ALDEADAVILA DE LA RIBERA |
| SÁNCHEZ CARRETERO FRANCISCO | C.S. F.VILLALOBOS (Sancti Spiritus) |
| SÁNCHEZ SÁNCHEZ TERESA | C.S. ALDEADAVILA DE LA RIBERA |
| SÁNCHEZ WHITE NICOLÁS | C.S. F.VILLALOBOS (Sancti Spiritus) |
| SANDIN PEREZ RAFAEL | C.S. SAN JOSE |
| SANZ SANTA-CRUZ FERNANDO | C.S. CAPUCHINOS |
| VELASCO MARCOS MARIA AUXILIADORA | C.S. ELENA GINEL DIEZ(TEJARES) |
| VICENTE LÓPEZ HORACIO MARCOS | C.S. ALDEADAVILA DE LA RIBERA |
| VICENTE SANTOS MANUEL SEBASTIAN | C.S. ALDEADAVILA DE LA RIBERA |
